# Stereotactic Radiotherapy in Combination with Immunotherapy in Treatment of Advanced Recurrent Squamous Cell Carcinoma of the Larynx

**DOI:** 10.3390/biomedicines11072067

**Published:** 2023-07-23

**Authors:** Paweł Polanowski, Aleksandra Nasiek, Aleksandra Grządziel, Agnieszka Pietruszka, Krzysztof Składowski, Katarzyna Polanowska

**Affiliations:** 1 1st Radiation and Clinical Oncology Department, Maria Sklodowska-Curie National Research Institute of Oncology, Gliwice Branch, Wybrzeże Armii Krajowej 15, 44-102 Gliwice, Poland; pawel.polanowski@gliwice.nio.gov.pl (P.P.); krzysztof.skladowski@gliwice.nio.gov.pl (K.S.); 2 3rd Radiation and Clinical Oncology Department, Maria Sklodowska-Curie National Research Institute of Oncology, Gliwice Branch, Wybrzeże Armii Krajowej 15, 44-102 Gliwice, Poland; 3 Radiotherapy Planning Department, Maria Sklodowska-Curie National Research Institute of Oncology, Gliwice Branch, Wybrzeże Armii Krajowej 15, 44-102 Gliwice, Poland; aleksandra.grzadziel@gliwice.nio.gov.pl; 4 Department of Clinical Oncology, Maria Sklodowska-Curie National Research Institute of Oncology, Cracow Branch, Garncarska 11, 31-115 Cracow, Poland; agnieszka.pietruszka@onkologia.krakow.pl; 5 Ophthalmology Department, St. Barbara Provincial Hospital No 5, Plac Medyków 1, 41-200 Sosnowiec, Poland; polanowskakatarzyna@gmail.com

**Keywords:** stereotactic radiotherapy, immunotherapy, squamous cell carcinoma, head and neck cancer

## Abstract

Squamous cell carcinoma (SCC) of the larynx in advanced stages is a challenging malignancy to treat with a high recurrence and death rate. An individualized approach to treatment is crucial in such patients. We present a 58-year-old male patient with SCC of the larynx in the T3N0M0 stage who was treated with concurrent radiochemotherapy. A total of 17 months after the radical treatment, the patient underwent a laryngectomy due to recurrence. A total of 11 months after the operation, local failure was diagnosed. In the next order, the patient received six cycles of palliative chemotherapy according to cisplatin 100 mg/m^2^ and 5-fluorouracil 1000 mg/m^2^. After three months, due to progression, Nivolumab-based immunotherapy was administered, ensuring disease stabilization. After the 56th cycle of Nivolumab, another progression was documented. The addition of stereotactic radiotherapy (18 Gy in three fractions) to immunotherapy led to significant regression of the disease and enabled the continuation of Nivolumab to the 70th cycle. The presented case demonstrates the usefulness of the combination of stereotactic radiotherapy with immunotherapy in prolonging the local control.

## 1. Introduction

Squamous cell carcinoma (SCC) of the larynx is one of the most common cancers of the head and neck region in European countries [1,2]. The predisposing factors for the development of the disease are alcohol consumption and tobacco smoking, whereas the most frequent symptom is persistent hoarseness. The standard treatment for patients in the early stages is radiotherapy (RTH) or surgery as an organ preservation procedure. Patients in advanced stages are qualified for radical treatment and require combined proceedings involving surgery, RTH, and chemotherapy. Approximately half of the patients with advanced SCC of the larynx will experience recurrence [3]. Platinum-based chemotherapy and immunotherapy based on Nivolumab or Pembrolizumab are the methods of life-prolonging therapy [3,4]. Stereotactic body radiation therapy (SBRT) plays a role in the treatment of isolated distant metastases or in cases of inoperable locoregional recurrence [5]. A combination of both treatments (SBRT and immunotherapy) may prove to be the most effective management in the future; however, there is currently no conclusive evidence to support this thesis. Some studies show that such management is an effective option for the treatment of recurrent SCC (although there is no consensus on total dose and fractionation) [6,7]. Others find no improvement in response or significant therapy impact [8]. This case report presents the comprehensive treatment management of laryngeal cancer with particular emphasis on the effective combination of immunotherapy and stereotactic radiotherapy.

## 2. Case Presentation

### 2.1. Diagnostic Imaging

We present a 58-year-old male patient with a 30-year history of smoking, reporting hoarseness for 6 months. Directoscopy visualized the tumor encompassing the right vestibular fold, laryngeal ventricle, and right vocal fold. In addition, vocal cord fixation of the right side was detected. A histopathological examination based on surgical biopsy revealed micro-focuses of SCC. Furthermore, a magnetic resonance imaging (MRI) scan of the head and neck was performed and showed a contrast-enhanced lesion measuring 25 × 15 × 17 mm and enlarged lymph nodes in group II bilaterally, measuring 14 mm on the right side and 13 mm on the left side. A fine needle biopsy was performed, which excluded the presence of neoplastic cells in the above lymph nodes. Additional examinations (abdominal ultrasound and chest X-ray) showed no distant metastases. The clinical stage was determined as T3N0M0.

### 2.2. Initial Treatment Characteristics

The patient’s case was presented at the Head and Neck Unit, where he was qualified for concurrent radiochemotherapy (CRT). A five-point head, neck, and shoulder mask with 5 mm bolus was fitted, and a computed tomography (CT) scan (3 mm slice thickness) without intravenous contrast and 18F-fluorodeoxyglucose positron emission tomography-computed tomography (18 F-FDG PET-CT) (3 mm slice thickness) in the supine position were performed. The dose prescription on the clinical target volume (CTV) involved bilateral neck lymph nodes in the range of groups II-V and group VI to a total dose of 50 Gy in 25 fractions (CTV1), larynx and II-III lymph nodes on the right side (groups at the most likely risk of metastasis—two lymph nodes on the border of groups II and III with increased 18 F-FDG maximum standardized uptake value SUV_max_ 3.9) to a total dose of 60 Gy in 30 fractions (CTV2) and laryngeal infiltration (gross tumor volume GTV, SUV_max_ 11.5) with a 5 mm margin to a total dose of 70 Gy in 35 fractions (CTV3). A 3 mm margin was added to the CTV, creating the planning target volume (PTV). Conventional treatment was applied on a C-arm linear accelerator (TrueBeam, Varian Medical Systems, Palo Alto, CA, USA). Radiation therapy was a three-stage treatment. In each of the stages, the VMAT (Volumetric Arc Therapy) technique with two 6X beams was used. Two full arcs in the range of 179–181° were executed in the first and second stages of treatment. In the third stage, a limited range of angles of 145–181° and 181–125° was implemented. The treatment plan was made on the basis of a 3 mm CT scan and AAA (Anisotropic Analytical Algorithm) calculation algorithm. Table 1 presents dose statistics for conventional radiotherapy. 

**Figure 1:**
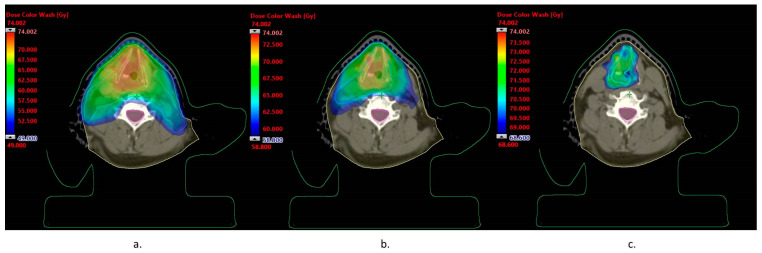
Dose distribution in conventional treatment: (**a**) isodose 49 Gy, (**b**) isodose 58.8 Gy, and (**c**) isodose 68.6 Gy.

Figure 1 shows the isodose distribution limited to doses of 49 Gy (a), 58.8 Gy (b), and 68.6 Gy (c), which is 98% of the prescribed dose of 50 Gy, 60 Gy, and 70 Gy, respectively. Dose washes are presented on the background of CTV 50 and PTV 50 (a), CTV 60 and PTV 60 (b), and CTV 70 and PTV 70 (c) target contours.

Concurrently, two cycles of Cisplatin-based chemotherapy 100 mg/m^2^ were administered. Leukopenia and neutropenia in grade II (Common Terminology Criteria for Adverse Events; CTCAE v5.0) were noted, resulting in a postponement of chemotherapy by 7 days. The application of prednisone 10 mg led to the normalization of hematological parameters. During the whole treatment, regular ear, nose, and throat (ENT) examinations were conducted. On the last day of radiotherapy, a 75% response to treatment was observed in ENT. Moreover, radiation reaction of the skin and mucosa in grade II (CTCAE v5.0) was registered.

In the first follow-up visit, one month after the end of the treatment, complete regression of the primary tumor in the flexible nasal endoscopy (FNE) was observed. Two months later, a CT scan of the head and neck was performed and reported the area of swelling with preserved slight residual infiltration. During the 16-month follow-up, regular ENT examinations showed no recurrence of the disease. A complete metabolic remission in the larynx was documented in the 18 F-FDG PET-CT scan in 10 months of follow-up.

### 2.3. Follow-Up Findings

A total of 17 months after the end of CRT, 18 F-FDG PET-CT showed a local recurrence of laryngeal cancer with cartilage involvement. Histopathological examination confirmed the recurrence of the disease, the patient was referred to the Laryngology Department for salvage treatment, and a total laryngectomy with selective lymph node dissection was performed. Histopathological examination showed SCC GIII, R0 resection with one close 4 mm margin. The pathological stage was defined as rpT4aN0. Eleven months after the operation, an MRI of the head and neck was performed, and the radiologist recognized local recurrence. Additionally, 18 F-FDG PET-CT highlighted the presence of the infiltration around the tracheostomy. Histopathological examination revealed SCC in specimens taken from the tracheostomy area.

The patient was presented to the Head and Neck Unit, where he was disqualified from surgery and qualified for first-line chemotherapy according to the PF regimen (cisplatin 100 mg/m^2^ on day 1 and 5-fluorouracil 1000 mg/m^2^ continuous infusion over 24 h for 4 days, repeated every 21 days) in the number of six cycles. Due to oropharyngeal dysphagia, percutaneous endoscopic gastrostomy (PEG) was set to ensure proper nutrition. After the third cycle of treatment, a CT scan was performed, where no radiological features of local recurrence were found. Moreover, the CT scan performed after the sixth cycle corresponded to the previous image. Treatment was complicated by peripheral polyneuropathy in grade I (CTCAE v5.0). Three months later, an infiltrated tracheostomy canal in the lower pole and on the left side was visualized in FNE. Specimens to histopathological examination were taken again and recognized infiltration of SCC.

### 2.4. Immunotherapy + SBRT

Due to progression, the patient was qualified for immunotherapy based on Nivolumab (240 mg i.v. every two weeks). Moreover, he was consulted with the endocrinologist before starting treatment, and levothyroxine (125 μg once a day) was prescribed due to hypothyroidism. No contraindications to starting immunotherapy were found.

The patient received immunotherapy regularly with imaging evaluation after a series of 7, 12, 18, 24, 31, 37, 41, and 47, where stable disease was confirmed. At the time of the 56th cycle, a CT scan showed an irregular, exophytic, 13 mm thick lesion in the tracheostomy area on the left side, indicating progression. Taking into consideration the patient’s good general condition, it was decided to continue immunotherapy. An 18 F-FDG PET-CT scan was performed and showed the active location of the primary disease at the level of the tracheostomy. The patient reported periodic bleeding from the lesion near the tracheostomy. Our team decided to perform stereotactic radiotherapy to the cancer infiltration (GTV) with a 3 mm margin (PTV) to a total dose of 18 Gy in three fractions and to continue immunotherapy in a 2-week cycle. The only symptom of late toxicity after almost 6 years from the end of conventional radiotherapy was skin atrophy on the neck.

Stereotactic treatment was based on a treatment plan made for two full 6FFF arcs in the VMAT technique. Calculations were made on 2 mm CT scans using the Acuros algorithm. To alter the dose distribution shallow under the skin, a 5 mm bolus was formed on the thermoplastic mask. The treatment was carried out on a C-arm accelerator (Edge, Varian Medical Systems, Palo Alto, CA, USA). Table 2 presents dose statistics for stereotactic radiotherapy. For other critical structures not included in the table, such as the brain, brainstem, chiasm, optic nerves, eyeballs, lenses, cochlea, parotid glands, and mandible, the maximum dose is below 0.3 Gy.

**Figure 2:**
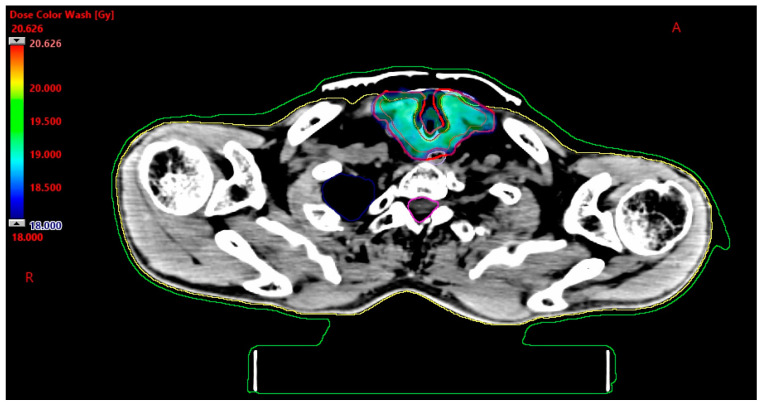
Dose distribution (isodose 18 Gy) in stereotactic radiotherapy.

Figure 2 shows a dose greater than and equal to the prescribed dose of 18 Gy. Contours of GTV, PTV, esophagus, spinal canal, and right lung are also presented.

**Figure 3:**
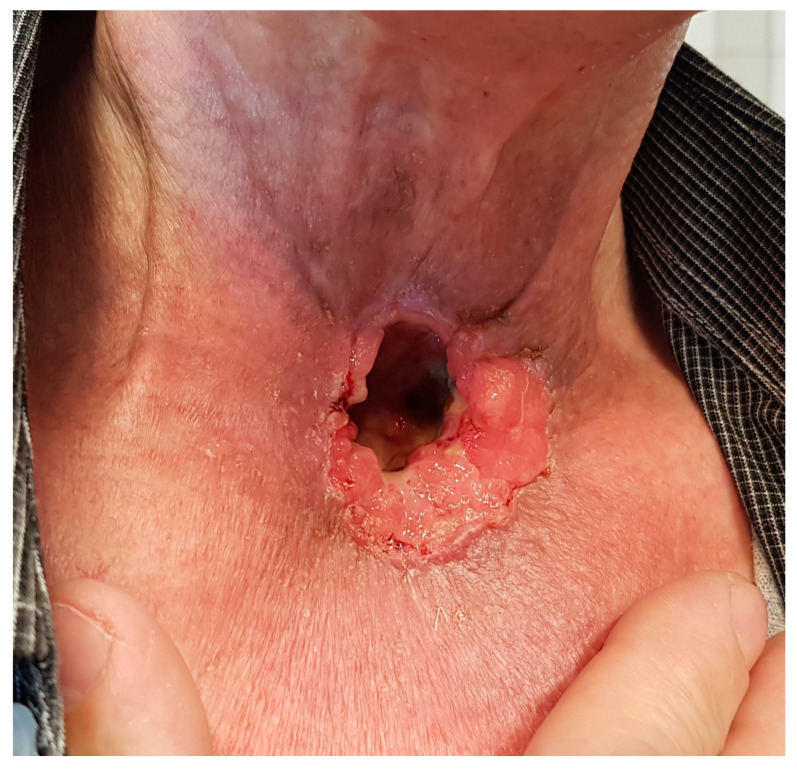
The infiltration before stereotactic radiotherapy.

**Figure 4:**
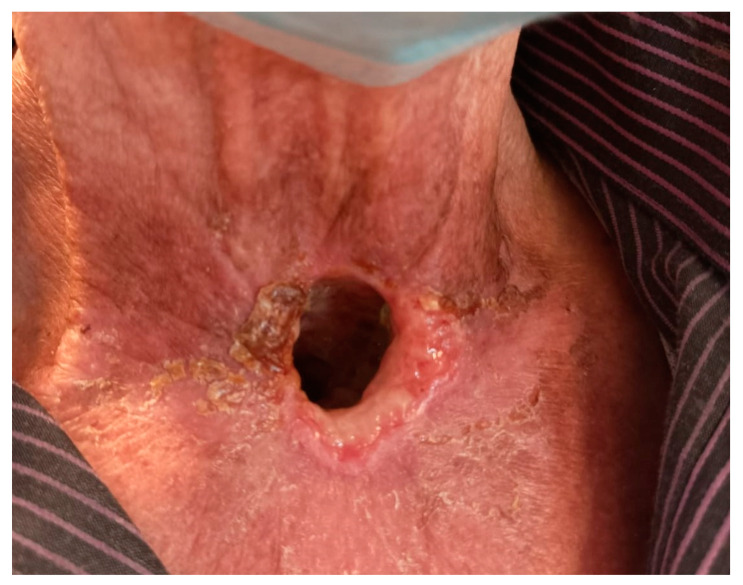
Regression of infiltration 6 weeks after stereotactic radiotherapy.

A total of 6 weeks after SBRT, the significant regression of cancer infiltration was confirmed in a physical examination and CT scan. The patient reported subsiding the bleeding. Figure 3 and Figure 4 show a comparison of the infiltration before and after SBRT combined with immunotherapy. This approach gave positive results and enabled the continuation of Nivolumab. At the time of the 65th cycle of Nivolumab, the patient reported symmetric shoulder joint pain of grade I (CTCAE v5.0). After a rheumatology consultation, there was no contraindication to continue treatment. Prior to the 66th cycle of immunotherapy, the patient was admitted urgently due to a fast-growing abscess in the chest wall. A CT scan of the chest revealed a multi-chamber abscess and reactive inflammatory changes located subclavian on the right side. A swab from the abscess was taken for bacteriological tests. Abundant colonies of Enterococcus faecalis, Serratia marcescens, Escherichia coli, Pseudomonas aeruginosa, Klebsiella pneumoniae, and Candida albicans were cultured. Polypragmasy (Biseptol 960 mg i.v. two times a day, Ampicillin 2g i.v. four times a day, Clindamycin 600 mg i.v. three times a day), analgesic, and anti-inflammatory treatment were initiated. Drainage of the abscess was performed using a vacuum-assisted closure (VAC-type) dressing. Two units of Red Blood Cell Concentrate were transfused due to grade III anemia (CTCAE v5.0). The applying management resulted in an improvement in the general condition and a decrease in inflammatory parameters. The patient was discharged home with a chest wound in the granulation stage.

**Figure 5:**
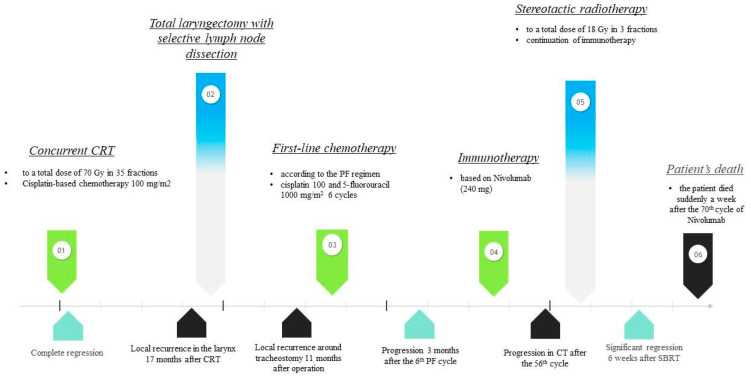
Graphical timeline of the patient’s treatment history.

After two months, a CT scan of the head, neck, and chest was performed. Thickening of tissues without contrast enhancement as residual infiltration or reactive changes after treatment around tracheostomy and post-inflammatory changes in the chest wall on the right side were visible. The abscess resulted in an almost three-month delay between the 65th and 66th cycle of immunotherapy. The 66–70th cycle of Nivolumab was given with acceptable tolerance. Unfortunately, the patient died suddenly a week after the last cycle of Nivolumab as a result of hemorrhage from the respiratory tract. The autopsy was not performed. Figure 5 shows a graphical timeline of the patient’s treatment history.

## 3. Discussion

The advanced stage of SCC of the larynx is usually characterized by an unfavorable prognosis. Historically, total laryngectomy was most commonly performed. However, today, a combination of chemotherapy and radiotherapy is used as a definitive therapy approach with organ preservation [2,3]. The long-term results of the Intergroup Radiation Therapy Oncology Group (RTOG) 91–11 study show that the use of concurrent CRT is an efficacious method. An analysis was conducted on 520 patients with SCC stage III or IV of the larynx who were randomly allocated to cisplatin/fluorouracil induction treatment followed by RTH (control group), concurrent cisplatin/RTH treatment, or RTH alone. The results of the study showed that a total of 148 patients underwent laryngectomy due to persistent or recurrent disease, with 32 of them belonging to the concurrent CRT group. Furthermore, it was observed that 80% of the laryngectomies occurred within the first two years, with 84 in the first year and 35 in the second year. However, this management resulted in better disease control and a prolonged rate of laryngectomy preservation than in the other groups [9]. Our patient underwent CRT, achieving complete regression of the disease. However, a recurrence was observed after one and a half years, similar to the findings in the aforementioned study. Due to the recurrence, the patient was qualified for salvage surgery. According to a systematic review conducted by Poorten et al., including studies from 2000 to 2021, it has been confirmed that salvage laryngectomy, following organ-preservation treatment, represents the optimal choice for achieving disease control and improving survival rates. The review included studies that described patients who underwent salvage treatment for residual and/or recurrent SCC of the larynx after initial concurrent CRT [10]. Our patient underwent a surgical procedure involving a laryngectomy and lymphadenectomy, but after 11 months, imaging indicated another recurrence of SCC. The patient was further qualified for palliative treatment according to the PF scheme. Based on Specenier’s review, this treatment regimen has become the most commonly used for patients with SCC of the head and neck region [11]. Moreover, Jacobs et al. compared the efficacy of using combined cisplatin and fluorouracil with their application as monotherapy in a group of 249 patients with recurrent head and neck cancer. The overall response rate was 32% in the group receiving cisplatin and fluorouracil and was higher compared to cisplatin alone (17%) or fluorouracil alone (13%) [12]. In context to the described case, three months after completing the sixth cycle PF, despite complete regression during chemotherapy, an infiltration was found in the FNE once again, and second-line treatment was required. In a global, open-label, randomized clinical trial, 361 patients with a disease progression of six months after completing platinum-based chemotherapy were qualified for treatment with either Nivolumab (3 mg/kg of body weight every 2 weeks) or standard systemic monotherapy (weekly administration of 40 to 60 mg/m^2^ methotrexate or 30–40 mg/m^2^ docetaxel or 250 mg/m^2^ cetuximab, after a first dose of 400 mg/m^2^). In the group of patients treated with Nivolumab, the median overall survival (OS) was 7.5 months. It had significantly longer effects compared with standard therapy, where the median OS was only 5.1 months [13]. In compliance with these results, our patient was qualified for the above-mentioned immunotherapy. However, a CT scan showed a progression of the disease after the 56th cycle of Nivolumab. In such a case, no clear treatment standards have been described. Our team decided to carry on immunotherapy and the addition of SBRT. Cengiz et al. presented the results of a study on the re-irradiation of locally recurrent, unresectable HNC using fractionated, frameless stereotactic radiotherapy. Nasopharynx, parapharyngeal localization, neck lymph nodes, oral cavity, maxillary, ethmoid or cavernous sinus, trachea, and esophagus were re-irradiated treatment sites. The applied dose was 30 Gy (range 18–35 Gy) in five fractions (range 1–5). A total of 37 patients were evaluated for response to treatment; 10 of them (27%) showed complete tumor regression, and 11 patients (29.8%) had a partial response. Definitive local control of the disease was achieved in 31 patients (83.8%). Tolerance of the treatment was good—one patient had mucositis GII, and two patients experienced dermatitis and mucositis, both GII [14]. The study highlights the usefulness of SBRT in salvage re-irradiation in the head and neck region. A notable example of the management of locally recurrent HNC is a study by Korean authors. Patients with different localizations of recurrence (nasopharynx, maxillary sinus, neck lymph nodes, skull base, nasal cavity, retropharyngeal lymph nodes, and orbit) were treated with CyberKnife fractionated stereotactic radiotherapy as salvage therapy, to a total dose of 18–40 Gy delivered in 3–5 fractions. Of the 44 sites evaluated, response assessment was performed in 35 of them, revealing 15 (42.9%) sites with a complete response and 13 sites (37.1%) with a partial response. A total of 13 patients developed acute complications GIII, and 3 patients experienced necrosis as a late effect. These results suggest that SBRT is an effective salvage treatment option for locally recurrent HNC; nonetheless, toxicity might be severe [15]. Radiotherapy shows potential in the induction of an anti-tumor immune response (influence on antigen presentation, modification of T-cell response, and the form of expression of immune checkpoint receptors), facilitating the enhancement of the effectiveness of immunotherapy. However, there is currently no consensus on the ideal dosage regimen to most effectively stimulate the immune system, but the use of 14–24 Gy delivered in two to three fractions with immunotherapy may be the optimal scheme [16]. McBride et al. performed a single-center, randomized phase II trial comparing Nivolumab versus Nivolumab in combination with SBRT in patients with at least two distant metastatic lesions of head and neck squamous cell carcinoma (HNSCC). The study enrolled 62 patients, with half randomly assigned to receive Nivolumab (3 mg/kg every 2 weeks), while the other half received Nivolumab at the same dosage plus SBRT (3 × 9 Gy). No significant difference in OS, disease progression-free survival, or duration of response was noted between groups. Severe toxicity (>GII) was in the range of 9.7–13.3%, also without significant difference [8]. On the other hand, Sari analyzed the group of 15 patients with locoregional recurrent or metastatic HNSCC. After two cycles of Nivolumab (3 mg/kg every 2 weeks), patients were treated with SBRT (24 Gy in three fractions at metastatic and recurrent sites). The six-month OS rate, the progression-free survival rate, and the local control rate at the site of SBRT were 93%, 86%, and 96%, respectively. Results after this treatment were encouraging because eight patients achieved a complete response, one showed a partial response, and only two patients experienced disease progression. Only one patient developed SBRT-related acute dermatitis GI. Dysphagia and pituitary insufficiency were developed as SBRT-related late effects in two cases, and one patient died due to skull base osteomyelitis as a late complication after SBRT [6]. In the context of our case, combined treatment, including stereotactic radiotherapy with immunotherapy, gave relevant regression and acceptable tolerance. The appearance of the abscess in the chest wall was probably related to a bacterial infection in the tracheostomy area as a result of insufficient hygiene, which was confirmed by the microbiological tests. The unequivocal determination of the cause of hemorrhage from the respiratory tract is difficult; most likely, it was the residual infiltration destroying large vessels. Re-irradiation toxicity seems less likely due to the six-year period between conventional and stereotactic radiotherapy.

## 4. Conclusions

This paper presents an example of a treatment pathway for advanced and recurrent SCC of the larynx when using radiochemotherapy, salvage surgery, palliative chemotherapy, immunotherapy, and re-irradiation with stereotactic radiotherapy. The combination of immunotherapy with an SBRT may enhance the response to treatment after progression during immunotherapy as a sole treatment providing good local control. Dose selection should be individual for every patient and take into account the dose distribution in prior radiotherapy.

## Figures and Tables

**Table 1:** Conventional radiotherapy—dose analysis.

	D_min_ [Gy]	D_max_ [Gy]	D_mean_ [Gy]
CTV 50	48.8	74.0	61.5
PTV 50	41.7	74.0	60.3
CTV 60	59.5	74.0	68.5
PTV 60	57.9	74.0	67.9
CTV 70	69.0	74.0	71.2
PTV 70	68.6	74.0	71.2
Spinal canal	0.0	44.8	17.8
Brainstem	1.0	20.8	3.0
Brain	0.4	11.7	1.4
Parotid gland left	4.9	57.7	25.1
Parotid gland right	4.2	64.0	26.5
Cochlea left	2.3	4.6	3.3
Cochlea right	2.2	3.4	2.7
Eye left	0.7	2.3	1.3
Eye right	0.7	1.8	1.1
Lens left	0.9	1.3	1.1
Lens right	0.8	1.1	1.0
Chiasm	1.5	1.9	1.6
Mandible	2.8	70.9	30.7
Thyroid	40.3	56.0	46.7
Lung left	0.0	47.7	1.7
Lung right	0.0	48.4	2.0
Vessels left	48.9	67.6	59.2
Vessels right	48.9	72.6	63.2

**Table 2:** Stereotactic radiotherapy—maximal dose analysis.

	D_min_ [Gy]	D_max_ [Gy]	D_mean_ [Gy]
GTV	17.0	20.6	19.0
PTV	16.2	20.6	18.9
Spinal canal	0.0	4.9	1.1
Esophagus	1.0	18.5	13.7
Lung left	0.0	13.3	0.7
Lung right	0.0	19.5	0.7
Vessels left	0.4	19.5	12.7
Vessels right	0.4	19.2	11.4

## Data Availability

Not applicable.
